# its2s: a Python package for two-stage interrupted time series analysis using machine learning

**DOI:** 10.64898/2026.07.02.26357175

**Published:** 2026-07-06

**Authors:** Lauren Blair Wilner, Joan A. Casey, Stephen J. Mooney, Vivian Do, Yiqun Ma, Tarik Benmarhnia, Arnab K. Dey

**Affiliations:** 1 Department of Epidemiology, University of Washington School of Public Health, Seattle, WA, USA; 2 Department of Environmental and Occupational Health Sciences, University of Washington School of Public Health, Seattle, WA, USA; 3 Department of Environmental Health Sciences, Columbia University Mailman School of Public Health, New York, NY, USA; 4 Huntsman Cancer Institute, University of Utah, Salt Lake City, UT, USA; 5 Scripps Institution of Oceanography, University of California San Diego, La Jolla, CA, USA; 6 Irset Institut de Recherche en Santé, Environnement et Travail, UMR-S 1085, Inserm, University of Rennes, EHESP, Rennes, France

## Abstract

When randomized controlled trials are infeasible, researchers may leverage natural experiments for causal inference. Interrupted time-series (ITS) designs compare observed post-event trends to counterfactual predictions from pre-event data. Two-stage ITS designs use flexible models to generate optimized counterfactual predictions in the first stage, then estimate intervention effects by comparing observed to predicted outcomes in the second stage. Fitting high-dimensional versions of these models is challenging, requiring systematic infrastructure to ensure rigor and reproducibility. In response, we developed its2s, an open-source Python package implementing the two-stage ITS design with machine learning. its2s allows users to specify an intervention date and training/testing periods, select among built-in model architectures (e.g., Prophet-XGBoost, NeuralProphet), and generate confidence intervals via moving block bootstrap, preserving temporal autocorrelation in residuals. its2s layers defaults, configuration files, and runtime overrides to support workflows ranging from rapid default implementations to highly tailored analyses. We validated its2s using two case studies: a simulation with a 12% policy effect, recovering the true effect as 11.77%, and an analysis of the 2021 Pacific Northwest heat dome, finding 53% excess injury mortality over the following three weeks. its2s provides a flexible, reproducible framework for ITSbased quasi-experimental research, lowering barriers to rigorous machine learning-based counterfactual modeling.

## 2 Introduction.

Causal inference is a central goal in health and policy research, where the causal effect is defined as the contrast between outcomes under treatment and outcomes under no treatment for the same units.^1,2^ A randomized controlled trial (RCT) is often considered the gold standard for internal validity, because well-conducted randomization creates two exchangeable groups and researchers control treatment and assessment, allowing them to infer that differences in the outcome can be attributed to the treatment. However, in multiple settings, including environmental exposures or social policies, RCTs are often infeasible or unethical.^3,4^ For example, researchers cannot assign climate disaster exposure to one group and a placebo to another – ruling out an RCT and making causal effects difficult to identify. In these cases, researchers may leverage natural experiments – events such as policy interventions, climate disasters, or disease outbreaks – whose timing is plausibly exogenous to factors driving the trends of a given outcome of interest, thus approximating randomization.^4^ Some quasi-experimental methods exploit this exogeneity to estimate causal effects from observational data, with different designs relying on different strategies to reconstruct the missing counterfactual. Interrupted time series (ITS) designs do so by modeling the outcome’s pre-event trend and attributing post-event deviations from that trend to the event of interest. ITS designs are particularly valuable when no credibly comparable control units are available – as is common for climate disasters such as wildfires and heatwaves, and for broad-coverage public health policies.^3,5,6^

ITS designs are applied to time series data: repeated measurements of an aggregate outcome -– such as weekly deaths or daily hospitalizations – collected at regular intervals in a population before and after an event. Beyond random variation, such series typically exhibit systematic temporal structure, including secular trends, seasonality, and temporal autocorrelation. Fundamentally, ITS designs compare observed post-event outcomes to counterfactual post-event outcomes – what would have been expected in the absence of the event.^6^ To estimate this counterfactual, the pre-event portion of the series is used to characterize the temporal structure, which is then projected into the post-event period. The most common implementation uses segmented regression to jointly model pre- and post-event trends as two separate linear segments.^5,7^ This generates estimates of both immediate level shifts (intercept changes) and longer-term trend shifts (slope changes) following the event.^6^

Besides traditional identification assumptions in causal inference (e.g. correct model specification, no spillover), using ITS designs requires additional assumptions including (1) the event timing is clearly defined, and (2) no other major events sharply affecting the outcome occur concurrently with the event of interest.^6^ Additionally, assumptions about the form of the counterfactual depend on the specific ITS design. In single-group ITS designs (sometimes called single ITS, or SITS), where no contemporaneous control series is available, the design assumes that when projected forward, the pre-event trend accurately represents what would have occurred in the absence of the event. Empirical work has shown that violations of this within-unit counterfactual assumption can be consequential.^8^

Beyond these design-level identification assumptions, the most common ITS implementation, segmented regression, has model-specific limitations. First, the researcher must pre-specify the functional form of the underlying trend (typically linear, with optional seasonal or polynomial terms) and the intervention effect (e.g., an abrupt level shift, a gradual slope change, or a lagged response) by hard-coding the corresponding dummy and interaction terms into the model.^3,5,9^ Selecting the functional form after inspecting the post-event data increases the risk of spurious conclusions and has been identified as a primary threat to ITS validity.^6,8^ Second, positively autocorrelated residuals – common in epidemiological time series – deflate standard errors and produce anti-conservative confidence intervals. While corrections such as Prais-Winsten or Restricted Maximum Likelihood with a Satterthwaite degrees-of-freedom adjustment can restore near-nominal coverage in longer series, no method reliably does so in the short series typical of applied ITS studies.^10,11^ Third, segmented regression returns a small set of global parameters (level and slope changes) rather than time-unit-specific excess estimates, limiting its utility for high-dimensional or pooled analyses. To address these limitations, several alternatives have been proposed, including Autoregressive Integrated Moving Average (ARIMA/ARIMAX), Bayesian ITS, and two-stage approaches with machine learning (ML).^7,9,12^

The two-stage ITS approach with ML is a flexible alternative to single-group ITS models that addresses the limitations specific to segmented regression.^9,13^ The “two-stage” label refers to a deliberate separation of tasks. In the first stage, a flexible ML model (e.g., a hybrid Prophet-XGBoost model) is trained on a subset of pre-event data, with the remaining pre-event data held out for testing and validation. Once a model is selected based on predictive performance, it is used to forecast the post-event period under the counterfactual scenario that pre-event trends would continue absent the event. In the second stage, researchers calculate the effect of the event by comparing the observed post-event series to this counterfactual. Separating these stages strengthens the analysis because (1) since the first-stage model never sees post-event data, the functional form cannot be tuned to the effect it is meant to estimate, and (2) the comparison is made at every time point, yielding a full trajectory of excess estimates rather than one or two global coefficients. Using an ML model further strengthens the credibility of the counterfactual, as flexible learners handle nonlinear trends, seasonality, and autocorrelation without pre-specification of the functional form. As with all single-group designs, the two-stage ML approach inherits the within-unit counterfactual assumption discussed above. However, it avoids the additional assumption required by controlled ITS designs that exposed and unexposed groups have parallel outcome trajectories over time. This is a meaningful advantage when no credible control unit exists. ML flexibility strengthens the credibility of the within-unit extrapolation, but it does not eliminate the assumption. Equally important, because model selection in stage 1 relies only on pre-event data, the impact model is never trained on (and thus never sees) post-event outcomes – structurally removing the data-driven specification bias that can compromise traditional ITS. The two-stage approach also yields time-unit-specific excess estimates rather than a single level or slope coefficient, allowing for richer characterization of dynamic and cumulative effects.^13^

Technical and computational barriers may limit uptake of the two-stage ITS approach with ML. Most applied researchers are experts in their respective fields but have limited specialized training in selecting cross-validation strategies, splitting data into training and testing sets, or choosing appropriate ML architectures for time series forecasting. ML approaches are also computationally demanding, and their complexity can make the underlying modeling assumptions difficult to state and test.^9^ Generating valid confidence intervals adds further complexity – methods such as moving block bootstrap (MBB) preserve temporal dependence by resampling contiguous blocks of residuals rather than individual observations, but require careful selection of block length, which is rarely straightforward.^9,13^ When researchers seek to evaluate multiple events, outcomes, or model specifications, tracking versions and ensuring reproducibility becomes challenging. While analytic code accompanying published applications of the two-stage ML ITS approach is publicly available,^13^ to our knowledge, no general-purpose, open-source software package implementing this design exists. Such a package could broaden adoption of the method and more reproducible research.

We developed its2s, an open-source Python package that provides a user-friendly implementation of the two-stage ITS approach with ML. The package couples built-in forecasting architectures with fully customizable default configurations. Reproducibility is supported through timestamped, versioned run directories and deterministic seeding across parallel batch runs. Here, we describe the methodological foundation for the two-stage ITS with ML design; detail the its2s package implementing this approach, and demonstrate its application through two case studies.

## 3 Methods.

To make the two-stage ITS with ML approach accessible to researchers, we developed its2s, an open-source Python package that implements this design with minimal barriers to entry. Users can provide as little as a time series dataset and specification of intervention/event date, leaning on built-in defaults and automation of model fitting, comparison, and effect estimation in its2s. Researchers can also leverage the full flexibility of its2s to customize every aspect of the workflow from data splitting and cross-validation strategies to hyperparameter tuning ranges and model selection. This flexibility allows researchers to conduct analyses ranging from rapid default implementations to fully customized workflows tailored to their specific research questions, all within a reproducible framework.

In stage 1, its2s splits the portion of the time series preceding the event into a training window and a held-out testing window. A ML model is trained on the training window to learn the outcome’s temporal patterns, then evaluated on the testing window to assess out-of-sample predictive power. Since the goal of this model is to forecast the unobservable counterfactual post-event trend, for which we do not know the truth, this evaluation of out-of-sample predictive power is essential. Flexible models can fit their training data well while generalizing poorly,^14–16^ so it is therefore vital to ensure that the counterfactual accurately represents the expected post-event period had the event not occurred. Train/test separation of this kind is the standard approach for evaluating predictive models in ML and time series forecasting; its2s makes it a structural, non-optional step of the ITS workflow.^16–19^

The selected model is then used to generate counterfactual predictions for the post-event period (or the holdout, in ML terms) - the expected time series had the event not occurred. In stage 2, its2s compares observed post-event outcomes to these counterfactual predictions to estimate the effect attributable to the event. Uncertainty is quantified using moving block bootstraps (MBB),^20^ which resamples blocks of residuals rather than individual observations to preserve temporal autocorrelation, following the approach in Dey et al.^13^ The statistical theory underlying the two-stage ITS approach is described in more detail in Dey et al.^13^

The two stages of the ITS are implemented in its2s through a small set of high-level functions organized around a simple core workflow. The primary entry point is run_single_its (), which executes the full pipeline for a single outcome series. This function accepts a time series dataframe, an event date, and optional specifications for the target, date, and covariate columns, the forecasting model, a YAML config (via path or runtime overrides), an output directory, and a random seed. The function then splits the pre-event period into training and test windows and fits and evaluates the selected forecasting model in Stage 1. Currently, this package supports the following ML model architectures for this model: (1) Prophet + XGBoost hybrid, (2) NeuralProphet, (3) Prophet then XGBoost, (4) ARIMA. As part of the Stage 1 fit, its2s automatically runs residual diagnostic checks via compute_diagnostics (), which evaluates the Ljung-Box statistic at pre-defined lags to detect residual autocorrelation^17^ and applies the Shapiro-Wilk test for normality.^21^ These diagnostics directly assess the validity of the moving-block bootstrap used in Stage 2, whose block length governs how much residual autocorrelation the resampling preserves and therefore the coverage of the resulting intervals. The block length may be fixed by the user (the default, specified in observations), or estimated automatically using the Politis and White plug-in rule^22^; in addition, its2s provides a diagnostic that traces the width of the event-window confidence interval across a range of candidate block lengths, letting the user confirm that the selected length falls in a region where interval width has stabilized. Because no block length restores nominal coverage when the Stage 1 residuals remain strongly autocorrelated, the Ljung-Box diagnostic serves to verify that the fitted model has adequately captured the temporal dependence before the bootstrap is applied. The pipeline returns a PipelineResult object containing fitted values, effect estimates, bootstrap confidence intervals, and model diagnostics. All results are accompanied by plot_counterfactual (), which produces a standardized figure showing the observed series, in-sample model fit, counterfactual prediction with confidence interval ribbon, and event marker. In the case where the same outcome is studied across multiple geographic units or sub-groups, users can use run_batch () to produce a list of series, parallelizing execution across cores and writing outputs to automatically versioned run directories that encode the run date and bootstrap simulation count for all combinations. Configuration at every step is managed through a YAML file loaded via load_config (), which specifies the split method, bootstrap parameters, and model hyperparameters; any parameter can be overridden at runtime via the config_overrides argument, and all overrides are documented in the model outputs, providing a reproducible specification that can be version-controlled alongside the data and code.

For researchers who want to interrogate model selection before committing to a final run, its2s also provides a set of pre-analysis tools: compare_models () runs all available model architectures on the same series and returns a ranked comparison table, tune_model () searches the hyperparameter space for a chosen model via Latin hypercube sampling, and time_series_cv () evaluates a single model’s out-of-sample performance across expanding folds independently of a full pipeline run.

We illustrate the package’s application in two case studies. First, we evaluate its2s using a simulated daily healthcare utilization series with a known ground-truth intervention effect – a synthetic +12% level shift imposed on March 1, 2025 – to assess how well the estimated counterfactuals recover the true effect. Second, we use counts of weekly injury deaths in Washington State from the US Centers for Disease Control and Prevention (CDC) to study the impact of the June 2021 Pacific Northwest heat dome, treating this extreme weather event as a natural experiment.

its2s is maintained on GitHub. All extended documentation can be accessed on the its2s website here.

## 4 Case studies.

Here we illustrate the utility of its2s in two case studies. Case study 1 leverages simulated data to recover the effect of a policy intervention on daily healthcare encounters. Case study 2 uses CDC Wonder data to quantify the impact of the 2021 Pacific Northwest heat dome on injury mortality in Washington state. The case studies are intentionally simple implementations of its2s, using predominantly default settings to illustrate the packages’ out-of-the-box utility.

### Casey study 2: simulated policy intervention

#### Background.

Case study 1 is a simulation representing a daily time series of healthcare utilization counts for a single geographic unit from January 2022 through December 2026, with a synthetic policy intervention on March 1, 2025. To generate these data, we imposed a baseline of ~800 visits per day with a moderate upward trend, annual seasonality (peak in early January), day-of-week effects (weekdays higher than weekends), and lower utilization on US federal holidays (~18% reduction). We sampled daily counts from a Poisson distribution. Beginning on the intervention date, we imposed a constant multiplicative +12% level shift on the rate. We then use its2s to estimate the intervention effect,, providing a ground truth against which to evaluate its2s.

#### Methods.

We use its2s with default settings and a Prophet + XGBoost model (default model) to fit the pre-intervention period. We reserved the last 20% of pre-intervention days for a testing set, using all remaining pre-intervention days as training data (default configuration). Our holdout was comprised of all post-event data (2025-03-01 through 2026-12-31). Uncertainty was generated using a MBB (default block length of 14 observations, equal to 14 days on this daily series). We estimated the change in utilization as the difference between the observed utilization count (from simulated time series) and the expected rate (counterfactual predicted from the model we fit on pre-intervention data).

#### Results.

**Figure 2:**
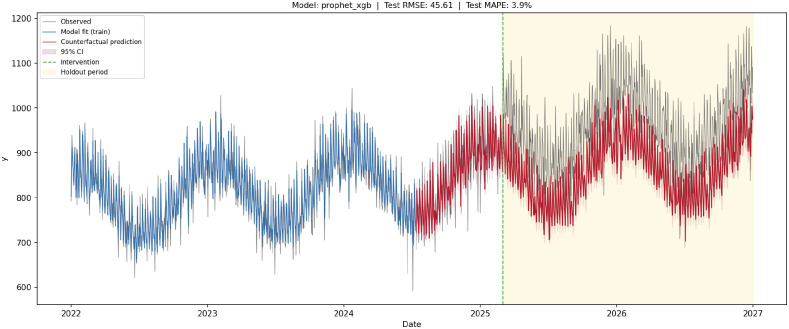
Case study 1: simulated daily data (grey), pre-intervention model fit (blue), and counterfactual prediction with uncertainty for the post-intervention holdout (red; Prophet + XGBoost). Dashed green line represents timing of event.

During the 671-day post-intervention holdout period (2025-03-01 through 2026-12-31), we estimated an 11.77% excess visit rate (95% eCI: 10.23%, 12.96%), approximately the true 12% level shift. The Prophet - XGBoost model fit the pre-event test time series reasonably well (*MAPE* = 3.9%) (Figure 2), facilitating the recovery of an effect size resembling the true effect.

### Case study 2: 2021 Pacific Northwest (PNW) heat dome

#### Background.

In the context of a warming climate, extreme heat is a growing public health concern linked to elevated mortality not only from cardiovascular and natural causes, but increasingly due to injuries (e.g., suicide, drowning, transportation accidents, or occupational accidents in construction or agriculture).^23–26^ Though historically the Pacific Northwest region of the US has a mild summer, in late June and early July, 2021, the region recorded its highest-ever temperatures due to a ‘heat dome’ where persistent high-pressure atmospheric system trapped and compressed.^27^ The unprecedented heat among a population with limited previous exposure to such temperatures and relatively little preparedness may have caused substantial excess mortality.^24,27^ In this case study, we treat the heat dome as a random shock and use its2s to apply an ITS analysis to study subsequent changes in weekly injury deaths in Washington State.

#### Methods.

We analyzed weekly counts of injury deaths in Washington State from January 2014 through July 2021 from CDC Wonder^28^ to estimate changes in injury mortality in the 3 weeks following the 2021 Pacific Northwest (PNW) heat dome (intervention date 2021-06-25; holdout weeks beginning 2021-06-26, 2021-07-03, and 2021-07-10). This analysis mirrors that of Casey et al. 2023 (^24^), who used ARIMA modeling. Our case study built a model using most of the default its2s settings. Given the strong seasonal temperature trends, we did not use default settings for the train/test split. Instead, we adjusted it to length the training period in order to ensure sufficient training data for the model to capture the seasonality sufficiently. We thus fit a new model on pre-event data (379 pre-event weeks and reserving 11 weeks (78 calendar days, 2̃.8% of pre-intervention observations) for out-of-sample testing immediately before 25 June 2021, a deviation from the default). We achieved a reasonable fit on these parameters, though future iterations could adjust the default settings further, too. The counterfactual time series was generated for the 3 weeks following the event, and uncertainty was quantified using MBB. The change in injury deaths was defined as observed minus expected (counterfactual) counts during the post-event holdout period.

#### Results.

**Figure 3:**
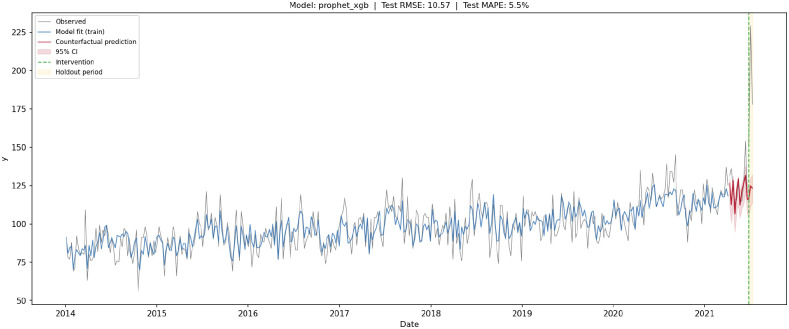
Case study 1: observed weekly injury deaths in Washington State (grey), model fit on pre-intervention data (blue), and counterfactual prediction with uncertainty for the 3-week post–heat-dome holdout (red; Prophet + XGBoost). Dashed green line represents timing of event.

During the 3-week holdout, we estimated 193 excess injury deaths (95% eCI: 179, 217) relative to the counterfactual, corresponding to a 53% excess (eCI: 49%, 60%). Excess injury-related mortality was highest in the middle week (week beginning 2021-07-03: 104 excess deaths [99, 118]), when observed counts reached 229 versus ~125 expected. The Prophet-XGBoost model fit the pre-event test time series reasonably well (*MAPE* =5.5%) (Figure 3).

This case study capitalizes on the random timing of the 2021 PNW heat dome to estimate changes in injury mortality attributable to the event. These results are comparable to Casey et al., who reported 159 excess injury deaths (95% CI: 122, 195) over a comparable 3-week window^24^. Our estimates in this ITS analysis are of similar magnitude and show that its2s can be applied in a few lines of code using default workflow settings. This analysis had many limitations – including lack of time-varying covariate adjustment and weekly aggregation, which may mask important daily changes in mortality – but demonstrated a user-friendly implementation of the package for a well-defined shock.

### What we learn from these case studies.

Together, these case studies show its2s applied with minimal API calls and largely default settings across two complementary settings – a simulated policy intervention in which the package closely recovered a known +12% level shift, and a real extreme-heat mortality application yielding plausible excess estimates. Both examples are illustrative, and are meant to highlight a single workflow for internal validation when ground truth is available. Real-world implementations may range from simple applications as demonstrated here to more complex ones that involve (1) covariate adjustment, (2) custom hyperparameter choices, (3) multi-model comparison frameworks, or (4) dynamic block length selection for estimating uncertainty using MBB.

## 5 Discussion.

The objective of employing a two-stage ITS with ML approach is to estimate the effects of an event or intervention by capitalizing on the timing of natural experiments. ITS can be applied to a wide range of natural experiments, including climate shocks (e.g., extreme heat events, wildfires, hurricanes), social or urban policies (e.g., alcohol control policies, protected bike lane) or healthcare policies (e.g., Medicaid expansion). The its2s software package allows users to readily implement this study design. The package provides a structured and documented tool for each decision point in the analytical process: time_series_cv () for evaluating out-of-sample performance, compare_models () for comparing model architectures, tune_model () for searching the hyperparameter space along options to use data-driven block-length selection for the bootstrap. its2s serves both straightforward use cases, allowing researchers to obtain results quickly with built-in defaults, and advanced use cases with customized pipelines.

Improving model performance – through model selection, covariate specification, or hyperparameter tuning – is the primary lever for strengthening the causal estimates produced by the two-stage ITS approach.^6,13^ However, achieving good test performance requires researchers to navigate a large and interdependent space of methodological decisions.^9,11,29^ First, researchers must determine the amount of pre-intervention data to reserve for testing, where too short a test window yields unstable performance metrics, while too long a window leaves too little training data – and the right balance depends on the total length of the pre-intervention series.^16,18^ Once the data are split, researchers need to choose from different model architectures, each with different assumptions about trend, seasonality, and residual structure.^6,9,30,31^ Evaluating candidates systematically requires time-series cross-validation, which introduces further structural choices (e.g., the number of folds, the validation window length, and the skip gap between folds needed to prevent information leakage into the held-out test period).^18,19^ Once a model is selected, searching the hyperparameter space adds another layer as each architecture exposes a distinct configuration space, and evaluating it systematically requires running many cross-validated fits whose cost grows with series length and model complexity.^9,31^ Further, when estimating uncertainty, for the MBB, the appropriate length should theoretically reflect the autocorrelation structure of model residuals - a structure that varies across series and model specifications.^9,20,22,32^ This means that a single fixed value for the block length is rarely justified, adding a further analytical decision after the model is fit.

These challenges scale sharply in typical epidemiological applications, where the same analytic steps often need to be repeated across multiple geographic units, age groups or other strata, and outcome types within a single study, and where sensitivity analyses may necessitate varying the training window, cross-validation specifications, or model specifications across an additional set of runs. By automating configuration management, batch processing, and version tracking, its2s eliminates much of the timeconsuming and error-prone infrastructure burden. This allows researchers to focus on scientific decision making – hyperparameter tuning, model selection, interpretation – rather than maintaining *ad hoc* hardcoded scripts and tracking systems.^9^

While its2s is well-suited for time series analyses with sufficient observations, a clearly defined event or intervention, and data over a relatively long time period (e.g., enough training data to span a full cycle of seasonality), the package has several limitations.

First, this package inherits all the limitations of an ITS design. The validity of the ITS design relies on effective counterfactual estimation, which requires an unverifiable assumption – that pre-event trends would extend into the post-event period absent the event of interest. While strong out-of-sample predictive power is a reasonable indicator of the model’s ability to generalize, it cannot ensure an accurate counterfactual. The design also assumes that no other shock coincides with the event of interest, which only the researcher, not the package, can check. Additionally, the ITS design is not a good fit for studies involving events or interventions with uncertain timing or prolonged duration. From a package perspective, the flexibility of its2s risks overfitting – requiring researchers to carefully test and validate models. Additionally, while its2s provides functionality to run large batches of different models, it does not handle model-specific customization automatically – this remains a decision for the research team. Finally, while its2s provides a computationally efficient framework, computational demands still scale substantially as datasets grow, batches increase, or hyperparameter grids expand.

When exposures cannot be randomized, natural experiments allow researchers to recover causal effects from abrupt exogenous shocks. If the timing of an event is assumed at-random with respect to other outcome determinants, using a population’s own pre-event trajectory can serve as the counterfactual for what would have occurred in the absence of the event – and the difference between the observed and expected outcome trajectories can be interpreted causally. Among the strongest methods for studying these natural experiments is the ITS design. The two-stage ITS with ML approach enhances this method by estimating a counterfactual that flexibly captures complex temporal and seasonal trends. This flexibility introduces methodological and computational barriers to entry that have limited its adoption. We introduce its2s as a solution, providing a standardized, reproducible, computationally efficient implementation of the method accessible to all skill levels. its2s expands the toolkit for leveraging natural experiments to conduct rigorous, reproducible causal inference – allowing researchers to rapidly and reproducibly quantify the impacts of exogenous shocks and policy interventions.

## Figures and Tables

**Figure 1:**
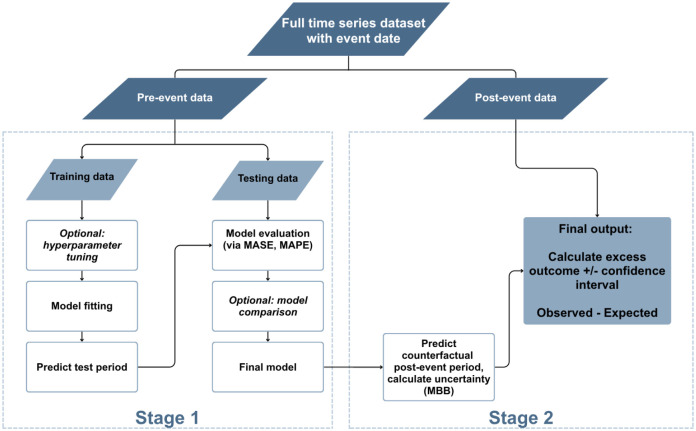
Description of its2s analytic workflow. Pre-event data is split into training and testing sets to build the Stage 1 model and predict the counterfactual time series. Stage 2 estimates the difference between the observed post-event time series and the counterfactual time series to estimate the causal effect of the event.

## Data Availability

All code and data are available in the GitHub repository.

## References

[1] Shadish WIlliam, Cook Thomas, and Campbell Donald. EXPERIMENTAL AND QUASI-EXPERIMENTAL DESIGNS FOR GENERALIZED CAUSAL INFERENCE. HOUGHTONMIFFLINCOMPAN, 2002.

[2] Hernan Miguel A and Robins James M. Causal Inference: What If.

[3] Kontopantelis E., Doran T., Springate D. A., Buchan I., and Reeves D.. Regression based quasi-experimental approach when randomisation is not an option: interrupted time series analysis. BMJ, 350(jun09 5):h2750–h2750, June 2015. ISSN 1756–1833. doi: 10.1136/bmj.h2750. URL https://www.bmj.com/lookup/doi/10.1136/bmj.h2750. 26058820 PMC4460815

[4] Craig Peter, Katikireddi Srinivasa Vittal, Leyland Alastair, and Popham Frank. Natural Experiments: An Overview of Methods, Approaches, and Contributions to Public Health Intervention Research. Annual Review of Public Health, 38(1):39–56, March 2017. ISSN 0163–7525, 1545–2093. doi: 10.1146/annurev-publhealth-031816-044327. URL https://www.annualreviews.org/doi/10.1146/annurev-publhealth-031816-044327. PMC6485604 28125392

[5] Bernal James Lopez, Cummins Steven, and Gasparrini Antonio. Interrupted time series regressionfor the evaluation of public health interventions: a tutorial. International Journal of Epidemiology, page dyw098, June 2016. ISSN 0300–5771, 1464–3685. doi: 10.1093/ije/dyw098. URL https://academic.oup.com/ije/article-lookup/doi/10.1093/ije/dyw098.

[6] Lopez Bernal J., Soumerai S., and Gasparrini A.. A methodological framework for model selectionin interrupted time series studies. Journal of Clinical Epidemiology, 103:82–91, November 2018. ISSN 08954356. doi: 10.1016/j.jclinepi.2018.05.026. URL https://linkinghub.elsevier.com/retrieve/pii/S0895435617314117. 29885427

[7] Schaffer Andrea L., Dobbins Timothy A., and Pearson Sallie-Anne. Interrupted time series analysis using autoregressive integrated moving average (ARIMA) models: a guide for evaluating largescale health interventions. BMC Medical Research Methodology, 21:58, March 2021. ISSN 1471–2288. doi: 10.1186/s12874-021-01235-8. URL https://pmc.ncbi.nlm.nih.gov/articles/PMC7986567/. 33752604 PMC7986567

[8] Baicker Katherine and Svoronos Theodore. TESTING THE VALIDITY OF THE SINGLE INTERRUPTED TIME SERIES DESIGN.

[9] Ma Yiqun and Benmarhnia Tarik. Interrupted Time Series Analysis in Environmental Epidemiology: A Review of Traditional and Novel Modeling Approaches. Current Environmental Health Reports, 12(1):50, December 2025. ISSN 2196–5412. doi: 10.1007/s40572-025-00517-3. URL https://link.springer.com/10.1007/s40572-025-00517-3. 41324811 PMC12669373

[10] Bottomley C, Ooko M, Gasparrini A, and Keogh RH. In praise of Prais-Winsten: An evaluation of methods used to account for autocorrelation in interrupted time series. Statistics in Medicine, 42(8): 1277–1288, April 2023. ISSN 0277–6715. doi: 10.1002/sim.9669. URL https://pmc.ncbi.nlm.nih.gov/articles/PMC10946734/. 36722328 PMC10946734

[11] Turner Simon L., Forbes Andrew B., Karahalios Amalia, Taljaard Monica, and McKenzie Joanne E.. Evaluation of statistical methods used in the analysis of interrupted time series studies: a simulation study. BMC Medical Research Methodology, 21:181, August 2021. ISSN 1471–2288. doi: 10.1186/s12874-021-01364-0. URL https://pmc.ncbi.nlm.nih.gov/articles/PMC8403376/. 34454418 PMC8403376

[12] Feder Kenneth A, Mojtabai Ramin, Stuart Elizabeth A, Musci Rashelle, and Letourneau Elizabeth J. Florida’s Opioid Crackdown and Mortality From Drug Overdose, Motor Vehicle Crashes, and Suicide: A Bayesian Interrupted Time-Series Analysis. American Journal of Epidemiology, 189(9): 885–893, September 2020. ISSN 0002–9262, 1476–6256. doi: 10.1093/aje/kwaa015. URL https://academic.oup.com/aje/article/189/9/885/5741085. 32077469 PMC7443765

[13] Dey Arnab K, Ma Yiqun, Carrasco-Escobar Gabriel, Han Changwoo, Rerolle François, and Benmarhnia Tarik. Two-stage interrupted time series analysis with machine learning: evaluating the health effects of the 2018 wildfire smoke event in San Francisco County as a case study. American Journal of Epidemiology, 194(10):2936–2944, July 2025. ISSN 0002–9262. doi: 10.1093/aje/kwaf147. URL https://pmc.ncbi.nlm.nih.gov/articles/PMC12527249/. 40663094 PMC12527249

[14] James Gareth, Witten Daniela, Hastie Trevor, and Tibshirani Robert. An Introduction to Statistical Learning, volume 103 of Springer Texts in Statistics. Springer New York, New York, NY, 2013. ISBN 978-1-4614-7137-0 978-1-4614-7138-7. doi: 10.1007/978-1-4614-7138-7. URL http://link.springer.com/10.1007/978-1-4614-7138-7.

[15] Cawley Gavin C and Talbot Nicola L C. On Over-fitting in Model Selection and Subsequent SelectionBias in Performance Evaluation.

[16] Tashman Leonard J.. Out-of-sample tests of forecasting accuracy: an analysis and review. International Journal of Forecasting, 16(4):437–450, October 2000. ISSN 0169–2070. doi: 10.1016/S0169-2070(00)00065-0. URL https://www.sciencedirect.com/science/article/pii/S0169207000000650.

[17] Bergmeir Christoph, Hyndman Rob J., and Koo Bonsoo. A note on the validity of cross-validation forevaluating autoregressive time series prediction. Computational Statistics & Data Analysis, 120:70–83, April 2018. ISSN 01679473. doi: 10.1016/j.csda.2017.11.003. URL https://linkinghub.elsevier.com/retrieve/pii/S0167947317302384.

[18] Hyndman RJ and Athanasopoulos G. Forecasting: principles and practice. URL OTexts.com/fpp3.

[19] Cerqueira Vitor, Torgo Luis, and Mozetič Igor. Evaluating time series forecasting models: an empirical study on performance estimation methods. Machine Learning, 109(11):1997–2028, November 2020. ISSN 0885–6125, 1573–0565. doi: 10.1007/s10994-020-05910-7. URL https://link.springer.com/10.1007/s10994-020-05910-7.

[20] Kunsch Hans R.. The Jackknife and the Bootstrap for General Stationary Observations. The Annals of Statistics, 17(3), September 1989. ISSN 0090–5364. doi: 10.1214/aos/1176347265. URL https://projecteuclid.org/journals/annals-of-statistics/volume-17/issue-3/The-Jackknife-and-the-Bootstrap-for-General-Stationary-Observations/10.1214/aos/1176347265.full.

[21] Shapiro S. S. and Wilk M. B.. An analysis of variance test for normality (complete samples). Biometrika, 52(3–4):591–611, December 1965. ISSN 0006–3444, 1464–3510. doi: 10.1093/biomet/52.3-4.591. URL https://academic.oup.com/biomet/article-lookup/doi/10.1093/biomet/52.3-4.591.

[22] Politis Dimitris N. and White Halbert. Automatic Block-Length Selection for the Dependent Bootstrap. Econometric Reviews, 23(1):53–70, December 2004. ISSN 0747–4938, 1532–4168. doi: 10.1081/ETC-120028836. URL http://www.tandfonline.com/doi/abs/10.1081/ETC-120028836.

[23] Romanello Marina, McGushin Alice, Di Napoli Claudia, Drummond Paul, Hughes Nick, Jamart Louis, Kennard Harry, Lampard Pete, Rodriguez Baltazar Solano, Arnell Nigel, Ayeb-Karlsson Sonja, Belesova Kristine, Cai Wenjia, Campbell-Lendrum Diarmid, Capstick Stuart, Chambers Jonathan, Chu Lingzhi, Ciampi Luisa, Dalin Carole, Dasandi Niheer, Dasgupta Shouro, Davies Michael, Dominguez-Salas Paula, Dubrow Robert, Ebi Kristie L, Eckelman Matthew, Ekins Paul, Escobar Luis E, Georgeson Lucien, Grace Delia, Graham Hilary, Gunther Samuel H, Hartinger Stella, He Kehan, Heaviside Clare, Hess Jeremy, Hsu Shih-Che, Jankin Slava, Jimenez Marcia P, Kelman Ilan, Kiesewetter Gregor, Kinney Patrick L, Kjellstrom Tord, Kniveton Dominic, Lee Jason K W, Lemke Bruno, Liu Yang, Liu Zhao, Lott Melissa, Lowe Rachel, Martinez-Urtaza Jaime, Maslin Mark, McAllister Lucy, McMichael Celia, Mi Zhifu, Milner James, Minor Kelton, Mohajeri Nahid, Moradi-Lakeh Maziar, Morrissey Karyn, Munzert Simon, Murray Kris A, Neville Tara, Nilsson Maria, Obradovich Nick, Sewe Maquins Odhiambo, Oreszczyn Tadj, Otto Matthias, Owfi Fereidoon, Pearman Olivia, Pencheon David, Rabbaniha Mahnaz, Robinson Elizabeth, Rocklöv Joacim, Salas Renee N, Semenza Jan C, Sherman Jodi, Shi Liuhua, Springmann Marco, Tabatabaei Meisam, Taylor Jonathon, Trinanes Joaquin, Shumake-Guillemot Joy, Vu Bryan, Wagner Fabian, Wilkinson Paul, Winning Matthew, Yglesias Marisol, Zhang Shihui, Gong Peng, Montgomery Hugh, Costello Anthony, and Hamilton Ian. The 2021 report of the *Lancet* Countdown on health and climate change: code red for a healthy future. The Lancet, 398(10311):1619–1662, October 2021. ISSN 0140–6736. doi: 10.1016/S0140-6736(21)01787-6. URL https://www.sciencedirect.com/science/article/pii/S0140673621017876. PMC7616807 34687662

[24] Casey Joan A., Parks Robbie M., Bruckner Tim A., Gemmill Alison, and Catalano Ralph. Excess Injury Mortality in Washington State During the 2021 Heat Wave. American Journal of Public Health, 113(6):657–660, June 2023. ISSN 0090–0036. doi: 10.2105/AJPH.2023.307269. URL https://pmc.ncbi.nlm.nih.gov/articles/PMC10186831/. 37023384 PMC10186831

[25] Parks Robbie M., Bennett James E., Tamura-Wicks Helen, Kontis Vasilis, Toumi Ralf, Danaei Goodarz, and Ezzati Majid. Anomalously warm temperatures are associated with increased injury deaths. Nature Medicine, 26(1):65–70, January 2020. ISSN 1078–8956, 1546–170X. doi: 10.1038/s41591-019-0721-y. URL https://www.nature.com/articles/s41591-019-0721-y. PMC6957467 31932800

[26] Burke Marshall, González Felipe, Baylis Patrick, Heft-Neal Sam, Baysan Ceren, Basu Sanjay, and Hsiang Solomon. Higher temperatures increase suicide rates in the United States and Mexico. Nature Climate Change, 8(8):723–729, August 2018. ISSN 1758–678X, 1758–6798. doi: 10.1038/s41558-018-0222-x. URL https://www.nature.com/articles/s41558-018-0222-x.

[27] Philip Sjoukje Y., Kew Sarah F., van Oldenborgh Geert Jan, Anslow Faron S., Seneviratne Sonia I., Vautard Robert, Coumou Dim, Ebi Kristie L., Arrighi Julie, Singh Roop, van Aalst Maarten, Marghidan Carolina Pereira, Wehner Michael, Yang Wenchang, Li Sihan, Schumacher Dominik L., Hauser Mathias, Bonnet Rémy, Luu Linh N., Lehner Flavio, Gillett Nathan, Tradowsky Jordis S., Vecchi Gabriel A., Rodell Chris, Stull Roland B., Howard Rosie, and Otto Friederike E. L.. Rapid attribution analysis of the extraordinary heat wave on the Pacific coast of the US and Canada in June 2021. Earth System Dynamics, 13(4):1689–1713, December 2022. ISSN 2190–4979. doi: 10.5194/esd-13-1689-2022. URL https://esd.copernicus.org/articles/13/1689/2022/.

[28] CDC. Underlying Cause of Death, 1999–2020 Request. URL https://wonder.cdc.gov/ucd-icd10.html.

[29] Ewusi Joycelyne E, Soobiah Charlene, Blondal Erik, Beyene Joseph, Thabane Lehana, and Hamid Jemila S. Methods, Applications and Challenges in the Analysis of Interrupted Time Series Data: A Scoping Review. Journal of Multidisciplinary Healthcare, Volume 13:411–423, May 2020. ISSN 1178–2390. doi: 10.2147/JMDH.S241085. URL https://www.dovepress.com/methods-applications-and-challenges-in-the-analysis-of-interrupted-tim-peer-reviewed-article-JMDH 32494150 PMC7231782

[30] Taylor Sean J. and Letham Benjamin. Forecasting at Scale. The American Statistician, 72(1):37–45, January 2018. ISSN 0003–1305, 1537–2731. doi: 10.1080/00031305.2017.1380080. URL https://www.tandfonline.com/doi/full/10.1080/00031305.2017.1380080.

[31] Chen Tianqi and Guestrin Carlos. XGBoost: A Scalable Tree Boosting System. In Proceedings of the 22nd ACM SIGKDD International Conference on Knowledge Discovery and Data Mining, pages 785–794, San Francisco California USA, August 2016. ACM. ISBN 978-1-4503-4232-2. doi: 10.1145/2939672.2939785. URL https://dl.acm.org/doi/10.1145/2939672.2939785.

[32] Lahiri S. N.. Theoretical comparisons of block bootstrap methods. The Annals of Statistics, 27(1), March 1999. ISSN 0090–5364. doi: 10.1214/aos/1018031117. URL https://projecteuclid.org/journals/annals-of-statistics/volume-27/issue-1/Theoretical-comparisons-of-block-bootstrap-methods/10.1214/aos/1018031117.full.

